# Multiple-System Atrophy in Long-Term Professional Painter: A Case Report

**DOI:** 10.1155/2012/613180

**Published:** 2012-06-19

**Authors:** Yusa Nagai, Riko Kitazawa, Miku Nakagawa, Munenori Komoda, Takeshi Kondo, Ryuma Haraguchi, Sohei Kitazawa

**Affiliations:** ^1^Division of Molecular Pathology, Ehime University Graduate School of Medicine, Shitsukawa, Ehime, Toon City, 791-0295, Japan; ^2^Department of Legal Medicine, Kobe University Graduate School of Medicine, 7-5-1 Kusunoki-cho, Chuo-ku, Kobe 650-0017, Japan

## Abstract

*Introduction*. Multiple system atrophy (MSA) is a rare and severe adult-onset, sporadic, and progressive neurodegenerative disorder. Here, we describe an autopsy case of MSA in a long-term professional painter. Although typical glial cytoplasmic inclusion (GCI) was not observed in a routine histological examination, strong *α*-synuclein immunostaining in the nucleus confirmed the diagnosis of MSA. *Case Presentation*. A 48-year-old Japanese man with a long occupational history of professional painter was sent to the emergency room, where he died of multiple organ failure. The patient had suffered tremors and inarticulateness at age 28, developed diabetes at 42 and was diagnosed with spinocerebellar degeneration at 46. A histopathological examination showed severe neuronal loss, gliosis, and tissue rarefaction in the paleostriatum, striate body of the substantia nigra, the pons, and the olivary nucleus of the upper medulla oblongata, intermediolateral of the spinal gray matter (sacral region). *α*-synuclein-positive GCI in oligodendroglia was occurred in the cerebral cortex, the midbrain, the medulla oblongata, and the spinal cord. These findings confirmed the presence of multiple-system atrophy (OPCA+SDS). *Conclusion*. Although the pathogenesis of MSA is still unclear, prolonged, and extensive exposure to organic solvents, together with a hyperglycemic morbidity attributed to diabetes, may have contributed to the onset and clinical course of the present case.

## 1. Introduction

Multiple system atrophy (MSA) is a rare and severe sporadic progressive neurodegenerative disorder of adult onset (average age at onset: 55–65 years), more frequent in men (1.3 : 1), and includes striatonigral degeneration (SND), Shy-Drager syndrome (SDS), and sporadic olivopontocerebellar atrophy (OPCA). The term MSA, comprising SND, SDS, and OPCA as one entity, was first introduced by Graham and Oppenheimer in 1969 [1]. MSA is now subclassified into two categories: associated, in varying proportions, with parkinsonism that is poorly responsive to levodopa therapy (MSA-P) and cerebellar dysfunction (MSA-C).

Glial cytoplasmic inclusion (GCI), designated as the histopathological hallmark of MSA in 1989 [2, 3], is an aggregated form of undigested *α*-synuclein characteristically observed in motor systems, the supraspinal autonomic sections, the putamen, the pallidum, and the lateral part of caudate nucleus, marking MSA as one in the category of *α*-synucleinopathy.

Here, we describe autopsy findings of MSA in a long-term professional painter. Although typical GCI was not observed in a routine histological examination, strong *α*-synuclein immunostaining in the nucleus confirmed the diagnosis of MSA.

## 2. Case Presentation

A 48-year-old Japanese man, a professional painter, was admitted to the emergency room. His past history revealed that at age 28, he consulted a general practitioner with the chief complain of tremors and inarticulateness, and followup showed intoxication from organic solvents. At age 42, he was diagnosed with diabetes and received regular outpatient treatment. Mitochondria-related neuropathy was ruled out through careful family history taking and a series of tests. By then he had started to use a wheelchair because of gait disorders, and complained of amnesia. At 46, the patient was diagnosed with spinocerebellar degeneration through extensive neurological tests. The symptoms gradually progressed to repeated episodes of aspiration pneumonia. The patient was found unconscious with cardiopulmonary arrest attributed to aspiration at supper, and despite attempts at resuscitation, he died of multiple organ failure. The autopsy was done 12 hours thereafter.

Postmortem examination revealed the body of a corpulent man, 167 cm tall, weighing 67.8 kg (BMI 24.8). Traumatic scars were noted in the left temporoparietal area, the precordium, and the right forearm. Marked disuse atrophy was observed in the extremities, especially in the legs. Observed also, besides severe lung congestion (left: 390 g and right: 520 g), were significant amounts of food debris and expectoration in the trachea and bronchi, with rubefaction and hemorrhagic change in the mucous membrane, and reflux esophagitis with numerous erosions in the mucous membrane of the stomach. The clinical diagnosis of repeated aspiration pneumonia was thus confirmed, and the direct cause of death was evidently due to suffocation by misswallowing. Furthermore, fatty liver (1815 g), pancreatic swelling (105 g), a moderate degree of aortic atherosclerosis, and marked accumulation of visceral fat reflected the longstanding history of type 2 diabetes mellitus. A neurogenic bladder with an expanded and trabeculated wall was also noted.

### 2.1. General Neuropathological Findings

**Figure 1:**
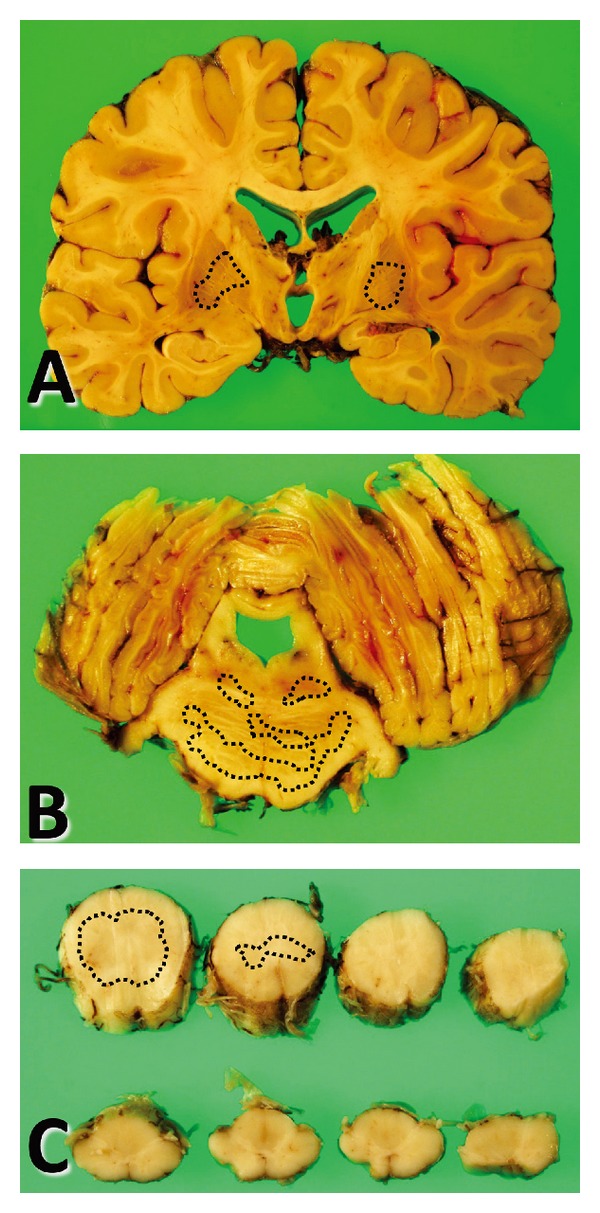
(A–C) Macroscopic findings. Severe atrophy of the paleostriatum together with dilatation of the lateral ventricle is evident in the coronal sections, while the thickness of the cerebral cortex is relatively intact (A). The brainstem, the gray matter of the cerebellar hemisphere (B), and the spinal cord (C) also show marked atrophy.

Gross examination of the brain (1300 g) revealed a highly atrophic brainstem, cerebellum, and spinal cord (Figures 1(B) and 1(C)), while the thickness of the cerebral cortex was almost intact (Figure 1(A)). Severe atrophy of the paleostriatum together with dilatation of the lateral ventricle was observed in coronal sections (Figure 1(A)). Additionally, the cerebellar hemisphere demonstrated fading in the gray matter (Figure 1(B)). On the other hand, no depigmentation of the substantia nigra or of the locus coeruleus was observed in sections of the brainstem (Figures 1(B) and 1(C)). 

**Figure 2:**
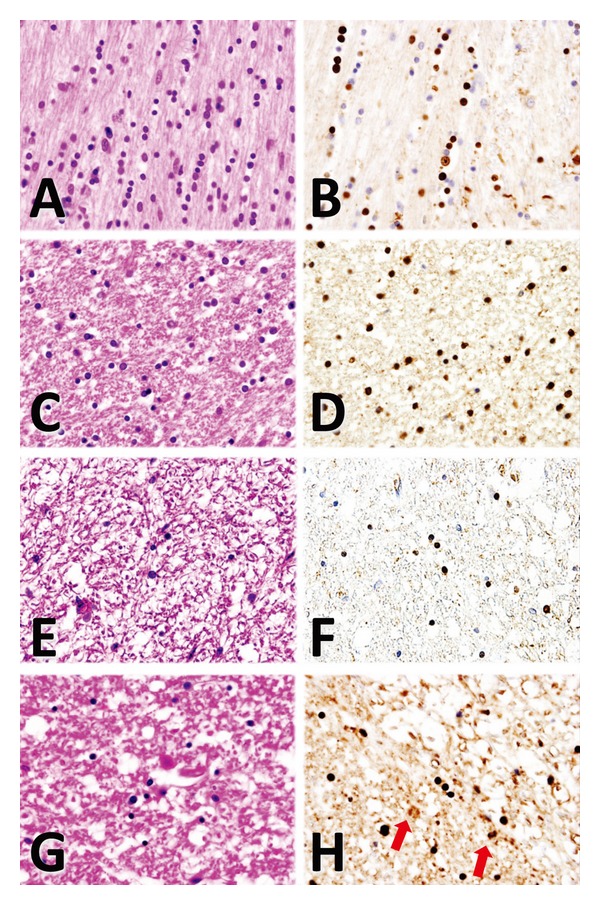
(A–H) Histological and immunohistochemical findings. Interfascicular oligodendroglia with halos in the cytoplasm and astrocytes are observed along with nerve fibers (A, HE staining). *α*-synuclein staining reveals numerous *α*-synuclein-positive glial cytoplasmic inclusions (GCI) in the oligodendroglia in the cerebral cortex (B). In the midbrain, the oligodendroglia and astrocytes show severe cytogenic edema (C, HE staining). *α*-synuclein-positive GCI in the oligodendroglia is also seen in the midbrain (D). The medulla oblongata shows extensive neuronal loss with scant oligodendroglial cells (E, HE staining), where *α*-synuclein is positive in the cytoplasma (F). Neuronal loss and gliosis are observed in the spinal cord (G, HE staining). Some *α*-synuclein-positive GCI in the oligodendroglia and astrocytes (arrows) are seen in the spinal cord (H).

A histopathological examination showed severe neuronal loss, gliosis, and tissue rarefaction in various areas, predominantly in the paleostriatum, the striate body of the substantia nigra, the pons, and the olivary nucleus of the upper medulla oblongata, intermediolateral of the spinal gray matter (sacral region) (Figure 2), all of which suggested multiple system atrophy (OPCA+SDS). 

The expression and localization of *α*-synuclein in the cerebral cortex, the midbrain, the medulla oblongata, and the spinal cord was determined by immunohistochemical analysis of the relevant formalin-fixed and paraffin-embedded specimens. Sections (4-*μ*m thick) were deparaffinized in xylene for 20 min (solvent refreshed at 10 min and 5 min), immersed in absolute ethanol for 10 min (solvent refreshed at 5 min), rehydrated in 90, 70% ethanol (5 min each), and finally placed in distilled water for 15 min (solvent refreshed every 5 min). The samples were then inactivated in 1 mM EDTA (pH 8.0) plus distilled water in a microwave oven for 15 min (high temperature for 5 min and low for 10 min) and cooled to approximately 35°C for 1 h. After liquid block treatment, the samples were immersed in phosphate buffered saline (PBS) for 15 min (solvent refreshed every 5 min). To observe the localization of *α*-synuclein, epitope specific rabbit anti-*α*-synuclein (spring bioscience, CA) was used as the primary antibody. After adding blocking buffer in a moisture chamber and incubating at room temperature for 1 h, the samples were washed 3 times for 5 min each in PBS, incubated again for 1 h with the secondary antibody (SC-2004, santa cruz biotechnology, inc., CA), diluted at 1 : 200 with PBS, and then immersed in PBS for 15 min (solvent refreshed every 5 min). The samples were then incubated with 3,3′-diaminobenzidine, tetrahydrochloride (DAB) for 15 min, washed in PBS, counterstained with hematoxylin, and observed and photographed under a microscope. The anti-*α*-synuclein antibody immunostained the glial and neuronal inclusions extensively, especially the cytoplasmic and nuclear inclusions in the oligodendroglia. *α*-synuclein-positive GCI in the oligodendroglia was occurred in the cerebral cortex, the midbrain, the medulla oblongata, and the spinal cord (Figures 2(B), 2(D), 2(F), and 2(H)). While gliosis and tissue rarefaction were observed in the midbrain, those in the locus coeruleus were relatively mild in the sections of the midbrain of the cerebellum level (data not shown).

## 3. Discussion

Patients with atypical parkinsonism are significantly more exposed to environmental toxins than are controls, and demonstrate a higher risk of disease onset associated with occupational exposure to organic solvents, plastic monomers and additives, pesticides, and metals [4]. Moreover, symptoms and neurological diseases are observed at a higher frequency in first relatives of MSA patients than in controls (23% in MSA cases versus. 10% in controls) [4]. Epidemiological studies in the French West Indies in 1999, on the other hand, implied an association between atypical parkinsonism and high consumption of tropical plants [5]. The European Study on Atypical parkinsonism in 2001 shows a significantly high risk of MSA in subjects with occupational exposure to various toxins, but a significantly lower risk among smokers, a factor often associated with a decreased risk of Parkinson's disease [6]. A more recent case-control study in the Aquitaine, France in 2004, shows a history of farming as significantly more frequent in MSA patients than in controls; it did not, however, reveal an association between occupational exposure to pesticides and MSA [7]. To date, on the other hand, no familial MSA case with a definite genetic background has been reported, nor has the genetic predisposition to MSA been established. Therefore, although exposure to environmental toxic substances may increase the risk of MSA, its pathogenesis is still unclear, and is simply defined as one of *α*-synucleinopathy characterized by the presence of GCI.

In the present case, the apparent risk factors for neurological manifestations were heavy exposure to organic solvents. Interestingly, an in vitro experiment has revealed that *α*-synuclein, originally in an unfolded form, is rapidly folded with an enhanced propensity to fibrillate in organic solvents [8]. Furthermore, by measuring hydrodynamic radii and adding glucose to solvents causes thorough collapse of *α*-synuclein [9]. The prolonged and extensive exposure to organic solvents together with the hyperglycemic condition attributed to diabetes may have contributed to the onset and clinical course of the present case.

## 4. Conclusion

Because most MSA cases lack a clear history of exposure to organic solvents with concurrent diabetes, some unknown genetic factors could define susceptibility to the disease. Accumulation of data from particular cases like the present one would be conducive to revealing clues in future studies.
